# Aligning organisational priorities and implementation science for cancer research

**DOI:** 10.1186/s12913-024-10801-x

**Published:** 2024-03-14

**Authors:** Stephanie Best, Karin Thursky, Mark Buzza, Marlena Klaic, Sanne Peters, Lisa Guccione, Alison Trainer, Jillian Francis

**Affiliations:** 1 University of Melbourne; Peter MacCallum Cancer Centre; Australian Genomics, Melbourne, Australia; 2 Peter MacCallum Cancer Centre; Royal Melbourne Hospital; University of Melbourne, Melbourne, Australia; 3 VCCC, Melbourne, Australia; 4 University of Melbourne, Melbourne, Australia; 5 Peter MacCallum Cancer Centre; University of Melbourne, Melbourne, Australia; 6 University of Melbourne; Peter MacCallum Cancer Centre, Melbourne, Australia

**Keywords:** Implementation science, Organisational priorities, Cancer, Theory informed, stakeholder

## Abstract

**Background:**

The challenge of implementing evidence into routine clinical practice is well recognised and implementation science offers theories, models and frameworks to promote investigation into delivery of evidence-based care. Embedding implementation researchers into health systems is a novel approach to ensuring research is situated in day-to-day practice dilemmas. To optimise the value of embedded implementation researchers and resources, the aim of this study was to investigate stakeholders’ views on opportunities for implementation science research in a cancer setting that holds potential to impact on care. The research objectives were to: 1) Establish stakeholder and theory informed organisation-level implementation science priorities and 2) Identify and prioritise a test case pilot implementation research project.

**Methods:**

We undertook a qualitative study using semi-structured interviews. Participants held either a formal leadership role, were research active or a consumer advocate and affiliated with either a specialist cancer hospital or a cancer alliance of ten hospitals. Interview data were summarised and shared with participants prior to undertaking both thematic analysis, to identify priority areas for implementation research, and content analysis, to identify potential pilot implementation research projects. The selected pilot Implementation research project was prioritised using a synthesis of an organisational and implementation prioritisation framework – the organisational priority setting framework and APEASE framework.

**Results:**

Thirty-one people participated between August 2022 and February 2023. Four themes were identified: 1) Integration of services to address organisational priorities e.g., tackling fragmented services; 2) Application of digital health interventions e.g., identifying the potential benefits of digital health interventions; 3) Identification of potential for implementation research, including deimplementation i.e., discontinuing ineffective or low value care and; 4) Focusing on direct patient engagement e.g., wider consumer awareness of the challenges in delivering cancer care. Six potential pilot implementation research projects were identified and the EMBED project, to support clinicians to refer appropriate patients with cancer for genetic testing, was selected using the synthesised prioritisation framework.

**Conclusions:**

Using a theory informed and structured approach the alignment between strategic organisational priorities and implementation research priorities can be identified. As a result, the implementation research focus can be placed on activities with the highest potential impact.

**Supplementary Information:**

The online version contains supplementary material available at 10.1186/s12913-024-10801-x.

## Background

Implementation of evidence based practice in healthcare is acknowledged to be challenging [1] and there is growing recognition of the research waste generated as investment in health research fails to make its way into practice [2]. In the US the annual total waste due to overtreatment or delivery of low-value care was estimated to be $75.7 billion to $101.2 billion [3]. With rising health care costs and changing consumer expectations it is essential that resources, including research activity, are optimised [4]. The research discipline of implementation science provides theories, models and frameworks that can help services better understand the barriers they face, navigate implementation by gathering evidence about which implementation strategies are likely to work, and in what contexts, and structure the research of implementation efforts more effectively [5, 6].

Implementation science provides a vehicle to investigate and address both top-down and bottom-up ways to approach implementation efforts. Top-down approaches focus on strategic planning and decision making and includes activities such as directives from senior management, financial incentives and resource allocation [7]. Clearly organisational leadership and resource management are key for successful implementation, but empirical evidence from a wide range of reviews is also clear that these factors are often not enough on their own to achieve lasting practice change. (https://epoc.cochrane.org/our-reviews) Bottom-up approaches centre on frontline activity including factors such as staff beliefs about an intervention, consumer engagement and alignment with professional identity [8]. A robust understanding of both context and implementation research is required to effectively examine and tackle some of the implementation challenges faced in the health and care setting. Better systems-wide integration of both top-down and bottom-up approaches has *potential* to promote a reduction in research waste with increased cost-effective use of resources and potential for impact. Measuring these aims is challenging [9], however work has been undertaken in the Basque health care system aligning top-down and bottom-up approaches as one component of developing systems-wide service transformation [10]. Top-down approaches included use of traditional formal approaches to planning and resource allocation while bottom up approaches place emphasis on clinical leadership.

Recognition of the potential for implementation science to address perceived gaps in care and to drive health services strategies has resulted in the recent creation by organisational leaders of implementation science roles embedded within healthcare services [11]. These roles may be funded directly by the healthcare organisation or indirectly through agreements with state governing authorities. Although the development of such roles can be a first step to build on work being undertaken by non-implementation science specialists, equally important is the development of a strategic plan e.g., a context-specific roadmap driven by key stakeholders, including for example, consumer needs, operational demand management and strategic priorities, to ensure implementation science activities align with the values and priorities of all end users including institutional leads, researchers and patients.

Building on a desire to minimise research waste and use implementation science in practice, our study sought to maximise the impact of implementation science resources, in the context of cancer care. We drew on implementation science theory and contextual expertise (clinical, consumer and organisational) to inform the direction of implementation science research. Engagement of key stakeholders is central to meeting the needs of the population. Stallings *et al* identify critical steps in the research process where a community’s engagement can be of value and includes the pre-research phase of establishing priorities [12]. The expert knowledge and opinion provided can result in the identification of a more relevant, meaningful and person centred outputs and outcomes for future implementation research [13]. Further, the inclusion of consumer voices supports diversification of views and helps identify what matters to patients leading, to more meaningful research [14, 15]. Given one of the aims of this study was to identify a pilot implementation research project, the involvement of stakeholders will be important to increase the acceptability and feasibility of subsequent activities [16].

Capturing key stakeholder views can garner multiple perspectives. Prioritisation is required to identify what should be addressed first. Research priority setting, i.e., seeking consensus about areas where research effort will have the widest benefits, is an essential step to maximising the impact of implementation science endeavours [4, 17]. To negate the traditional emphasis on investigator-driven research [18] various methods have been used for priority setting in the organisational context including the Delphi technique, expert panels, consensus conference, ranking or voting, surveys, focus groups, and interviews [19]. Although no preference is given to any particular method Fadlallah et al’s [19] scoping review suggests consideration is given to the methods used where the emphasis is placed on consumer engagement, equity, a specific field of research, or resource availability. It is vital the process is context-relevant, transparent and with explicit engagement of key stakeholders such as those in receipt of, or responsible for delivery or the organisation of cancer care including consumers, clinicians and organisational leads. We sought the views of key stakeholders through semi-structured interviews, drawing on theory to guide the process.

### Aims and objectives

To optimise the value of embedded implementation researchers and resources, the aim of this study was to investigate stakeholders’ views on opportunities for implementation science research in a cancer setting that hold potential to maximise impact on care. The research objectives were to: 1) Establish stakeholder and theory informed organisation-level implementation science priorities and 2) Identify and prioritise a test case pilot Implementation research implementation project.

## Methods

### Context

The Peter MacCallum Cancer Centre (PMCC) is a leading specialist cancer care organisation with a strong global reputation for best practice research and patient care. The Victorian Comprehensive Cancer Centre Alliance (VCCC Alliance) is a multidisciplinary alliance of 10 leading research, academic and clinical institutions working collaboratively to accelerate cancer research, knowledge and expertise to benefit the Victorian community. Both based in Melbourne, Australia, the PMCC and VCCC Alliance recently invested in their existing implementation science infrastructure with the appointment of a senior research lead for implementation science - providing an opportunity to consolidate effort and set the implementation science research agenda.

### Study design

To capture the rich and varied perspectives of a wide range of stakeholders, we undertook a qualitative study with iterative data collection and analysis. The target population were those with ‘decisional authority’ playing a key role at either PMCC or VCCC Alliance. We established a cross organisational working group of implementation science and organisational health service research leads (JF, KT, MB, SB with support from operational staff at PMCC and VCCC Alliance and implementation researchers) to guide the study. The embedded implementation scientist, co-located in the PMCC/VCCC Alliance, enabled the exploration of contextually determined priorities for this study.

### Participants and recruitment

Participants were affiliated with the PMCC or VCCC Alliance. Inclusion criteria included: PMCC/VCCC Alliance members with either:

- a formal leadership role e.g., Head of Department, clinical research leads and clinical education leads;
- active PMCC/VCCC Alliance researchers e.g., submitting grants to federal grant opportunities (e.g., Australian NMHRC, MRFF) or more than two publications in the last year;
- consumer advocates working with PMCC/VCCC Alliance.

Exclusion criteria comprised: PMCC/VCCC Alliance staff who did not hold a formal leadership position and were not research active; and individual consumers. A convenience sample was identified from the target group to include participants from a range of regional sites and clinical specialities. Potential participants were identified by the working group and nominations were also sought from the wider PMCC Department of Health Services and VCCC Alliance operational staff using the inclusion criteria. All those nominated were invited to interview by email (SB). The invitation included participant information and a request to contact the researcher if they were interested in participating. We actively sought a heterogeneous population demanding a large sample size [20].

### Data collection and procedures

We used semi-structured interviews to capture data to gather the breadth of participants’ perceptions [21] on opportunities for implementation research in a cancer setting in order to maximise impact on care. Focus groups would have provided another data collection approach [22] but identifying available time in participants diaries did not lend itself to this method in this study. The interview schedule was devised drawing on Patey et al’s framing of implementation science activity (Supplementary file I) [23] e.g., clarity about the type implementation challenge – is it a new intervention, slow uptake etc., and a collaboration guide (Best, S., Peters, S., Guccione, L., Francis, J., Klaic, M. Implementation science ready? A guide to frame discussions between clinicians and implementation scientists [in preparation])e.g., evidence that the implementation challenge needs addressing [24]. The interview questions guided an exploration of implementation problems, potential solutions e.g., What are the sticky or thorny implementation issues that are resistant to change? How do you know this is a problem – what evidence do you have about the care gap? Semi-structured interviews were conducted online at a time of the participants’ choosing between August 2022 and February 2023. Interviews were not audio recorded in order to establish trust [25] and notes were made at the time of the interview. Post interview, a summary (template - Supplementary file II) was emailed to interview participants for their review and revision as necessary. Ethical approval was provided via the Peter MacCallum Cancer Centre Human Ethics Committee No: 22/92L and individual informed verbal consent was requested and provided at the start of each interview by each participant.

### Data analysis

The interviews were conducted over several months and so data analysis commenced before data collection was completed. As a result, completed interviews iteratively informed sequential drafts of data analysis which in turn informed subsequent interviews. Before analysis commenced, identifiable participant data were replaced with a unique re-identifiable code. Data analysis comprised of three stages: 1. Thematic analysis to identify priority areas for implementation research, 2. Content analysis to identify potential pilot implementation research projects and 3. Prioritisation.

1. Thematic analysis: Interview data were analysed thematically to detect recurrent themes. (Braun & Clarke, 2020) There were several stages: first, immersion in the data through reading and re-reading the interview data (SB); concurrently, clean data were shared at the fortnightly implementation planning meetings (JF, KT, MB, SB); initial, transient codes were identified and shared before firmer themes were compiled (JF, KT, MB, SB). We sought concept density rather than data saturation, seeking depth and diversity of views. (Nelson, 2016)
2. Content analysis: Similar to the thematic analysis, the interview transcripts were read and re-read (SB) with clean data shared at the fortnightly implementation planning meetings (JF, KT, MB, SB). Our level of analysis was a potential project, rather than themes. The interview data were screened to identify potential projects by the cross-organisation Project Working Group and subsequently prioritised (JF, KT, MB, SB).
3. Prioritisation: We used a synthesis of two frameworks (Table 1) a. Organisational priority setting [26] and b. APEASE framework [27]. These two frameworks were selected to provide an organisational and implementation science perspective as an existing organisational and implementation science prioritisation tool was not available. The domains within the two frameworks were compared (JF, KT, MB, SB) with criteria combined where commonality was detected by the team (see Table 1). Discussions extended across fortnightly implementation meetings. Oversight was provided by organisational research leads.

**Table 1:** Organisational priority setting [26], APEASE frameworks [27] and synthesis of the two frameworks

**Organisational priority setting**	**APEASE framework**	**Synthesised frameworks**
Strategic fit Alignment with internal and external directives	Acceptability	Alignment with organisation priorities
Clinical impact	Effectiveness and cost-effectiveness Side-effects / Safety	Potential for clinical impact
Community needs	Equity	Potential to narrow equity gap
Resource implications	Practicability Affordability	Resource implications
Partnerships (external) Interdependency (internal) Academic commitments – i) education ii) research		Potential for capacity building/networking

 The Project Working Group assigned ratings with consensus to each of the potential implementation research projects to prioritise which one would be selected as the exemplar project. Alignment with each of the synthesised criteria were graded as, red – poor alignment; amber – potential alignment; and green – good alignment. The process of rating was undertaken by the study and shared with the VCCC Alliance Distributed Leadership team (comprising of clinicians, consumers and operational staff) for validation.

## Results

### Participant characteristics

In total 31 people were identified to participate in the study. A peak in COVID-19 cases resulted in four people being unavailable for interview. As a result, 27 people were invited to interview and 25 responded. Of the 25 participants the majority were physicians/surgeons (*n*=19), two were nurses, two were consumers and two were in operational roles (e.g., equity and diversity leads). Many participants held several roles and Table 2 provides the breakdown by inclusion criteria and the role code used to report the themes.

**Table 2:** Participant characteristics

**Role description**	**Role code**	**Participants**
Formal leadership role e.g., Head of Department, clinical research leads and clinical education leads;	L	20
Active PMCC/VCCC Alliance researchers e.g., submitting grants to federal grant opportunities (e.g., NMHRC, MRFF) or more than two publications in the last year	R	19
Consumer advocates working with PMCC/VCCC Alliance.	C	2

N.B. Participants could hold several roles

Key: L - leadership, R - research, C - Consumer

### Establishing implementation research priorities - themes

Four themes were identified: 1) Integration of services to address organisational priorities; 2) Application of digital health interventions; 3) Identification of potential for implementation research, including deimplementation and; 4) Focusing on direct patient engagement. Here we elaborate on each theme providing challenges and, where identified, the opportunities alongside associated exemplar notes.

#### Integration of services to address organisational cancer priorities

Participants identified a range of strategic issues that could form the focus of an implementation study. These issues included the distribution of cancer services, future planning for delivery of cancer care and the importance of building relationships across clinical and research fields. Several participants reported the importance of addressing fragmented services and specialities located across multiple sites. At times this related directly to cancer care and others referred to more holistic care, “*Siloed working (between and within hospitals). This is challenging for everyone but in particular older people and people with mental health issues*” Interview 12C. There was concern from some about projects being undertaken with a lack on onward planning. Participants noted a lack of cohesion with implementation planning from the creation of concepts to improve cancer care then a lack of focus on the implementation in practice; “*Development of policies and frameworks which don’t then get implemented – they lack an implementation plan and evaluation*” Interview 12C. Solutions to addressing strategic challenges of integration of cancer services centred on social influences. Participants were keen to see time invested in building cross organisation relationships, “*build relationships, bring people together including consumers, clinicians from other fields to cross fertilise ideas and humanise others*” Interview 11C and to see research, education and clinical care brought together, “*Integrating research, teaching and patient care – there are many great researchers who are not necessarily great clinicians*” Interview 8LR.

#### Application of digital health interventions

Participants reported the use of digital health interventions in cancer care as a potential implementation study. The upswell of digital health interventions for delivery or as an adjunct to cancer care was commonly reported. However, some participants voiced frustration about the use of digital health interventions to date reporting early enthusiasm with digital health projects that were not seen to benefit patient care, “*Getting people engaged with [digital] change projects – sacrificing time short term to reap benefits longer term. To some extent it is understandable as people have been promised results that then didn’t materialise*” Interview 2LR. Equally, many recognised the potential of digital health interventions and suggested a range of ideas to address previously poorly informed digital health projects including, “*A short term bidirectional [digital] platform to mirror the patient journey.” This could provide the opportunity to reassure patients who are (within normal limits) feeling unwell and accelerate care for those who need attention*” Interview 18R and, “*Establishing the use of data hub to capture clinical outcomes in regional settings*” Interview 4R.

#### Identifying potential for implementation research, including deimplementation

Several participants discussed a focus on implementation research in the context of cancer care, as opposed to a specific cancer care service delivery challenge. Implementation research topics included the potential for deimplementation i.e., discontinuing ineffective or low value care [28] and features to consider with implementation research. The call for deimplementation research was typified by Interview 3R “*There is interest in low value care, deimplementation and why people don’t accept new evidence*”. With key drivers in deimplementation also reported, “*Deimplementation requires i. clinical leadership, ii, robust evidence, iii, feasible data collection*” Interview 17LR. Important features for implementation research included involvement from all craft groups such as nurses, “*Currently there is no real expectation of nurses to be involved or leading implementation project from organisation*” Interview 10LR. Some expressed the need to consider the ongoing sustainability of implementation research, “*Embed implementation research into day to day practice*” Interview 19/20R.

#### Focusing on direct consumer engagement

While many of the participants raised the importance of a focus on consumer need and engagement with consumers for implementation research in cancer care, some highlighted the importance of activity that was centred on patients’ and families needs. Identifying consumer needs was reported as a priority and not assuming clinicians or organisational leaders know what consumers want from their cancer care, “*Ask patients/families what their expectations are? Are they realistic? If not, identify who will have the difficult discussion with them*” Interview 8LR and, “*Need to partner with consumers and this engagement must not be tokenistic*” Interview 12C. At times to the focus was on individual patient needs, “*Discharge decisions need to be patients’ values centred and to identify what trade-offs they are happy to make, especially those with poor prognosis*” Interview 8LR. While others considered wider population awareness of the finite resources available for cancer care, “*Lack of community understanding of challenges facing healthcare*” Interview 12C.

### Implementation research projects identified

In total, six potential pilot implementation research projects were identified from the interviews (Table 3). Projects included some that were focused on staff e.g., upskilling the nursing workforce through an implementation fellowship programme, while others were patient focused e.g., investigating delayed presentation for cancer care for people living at a distance from a specialist cancer centre.

**Table 3:** Potential pilot Implementation research projects identified at interview

**Implementation research project title**	**Short description**
Tapping into the potential of the cancer nursing workforce in implementation research	This study targets the largest craft group in cancer health care and looks to trialling an implementation fellowship programme. Cancer nurses interested in taking part will be recruited and supported to design, deliver and evaluate their implementation project through a mix of specialist implementation researcher input, directed learning and an implementation nurse community of practice. Emphasis will be placed on narrowing the equity gap.
Investigating delayed cancer presentations for people living at a distance from a specialist cancer centre	Clinical outcomes are poorer for people with cancer who live at a distance from specialist cancer care services [29]. This study would capture the views of people diagnosed with cancer living remotely from a metropolitan specialist cancer centre such as PMCC, to identify the challenges and importantly what facilitators would support people to present early. This study could adopt an equity lens and target people in priority populations
Exploring cancer clinicians’ perspectives on deimplementation of low value care	Deimplementation of any clinical care is challenging, even when it has been shown to provide low value care. There are multiple reasons for this including consumer views and expectations and clinicians’ perspectives. This study would focus on clinicians’ perspectives on shifting care from a predominantly surgical model to conservative care in prostate cancer, where appropriate; and follow up care e.g., over investigation and unnecessary ordering of investigations.in their clinical specialty.
Initiation of virtual multidisciplinary team meetings MDTs for rare cancers	It is challenging for clinicians and patients to access support and expertise for some rare and less common cancers, e.g., penile and testicular cancer, in regional locations. This study would investigate the impact of a virtual MDT to support clinicians caring for people with rare and less common cancers. Using a process evaluation approach this study would capture clinicians’ perspectives of the virtual MDT and the impact.
Examining and addressing the barriers and enablers to quality data entry to clinical registries for cancer services	Despite recognition of the importance of clinical registries there are challenges in getting good quality (i.e., clean, accurate and reliable) data inputted. Some of this can be attributed to lack of time but the challenges go deeper than this. Studies show adherence to clinical guidelines improves quality of care [30]. This project aims to examine the influences on registry data entry, including feedback mechanisms, to identify interventions to promote improved quality data entry.
EMBED - supporting clinicians to offer genetic testing for rare cancers	The Victorian Cancer Registry is notified when a person is diagnosed with cancer. For some cancers there is value in then conducting a genetic test which can sway clinical management and also have implications for other family members. At present the number of people who are tested is low and does not represent the diverse population of Victoria. This project will establish a mechanism to inform clinicians of the need for testing in specified rare cancers (following agreement across the Victorian Familial Cancer Centres, [FCC]). We will develop a ‘test and tell’ intervention to support clinicians to offer genetic testing to their patients or refer to the FCC for testing.

### Prioritisation

The Project Working Group prioritised the potential projects identified from the interviews using the synthesised framework from Table 1. Results can be found in Table 4. The project with the most prioritisation criteria coded green, the EMBED project, was designated the highest priority. This project aims to widen the diversity of consumers having access to genetic tests for selected rare or less common cancers through active identification of relevant consumers and prompting the need for referral. The EMBED project provides an opportunity for organisational leads to input into this study and through this engagement to build their implementation science capacity. The importance of the consumer voice was recognised and the study will include a consumer group to inform how the project progresses and influence data analysis.

**Table 4:**
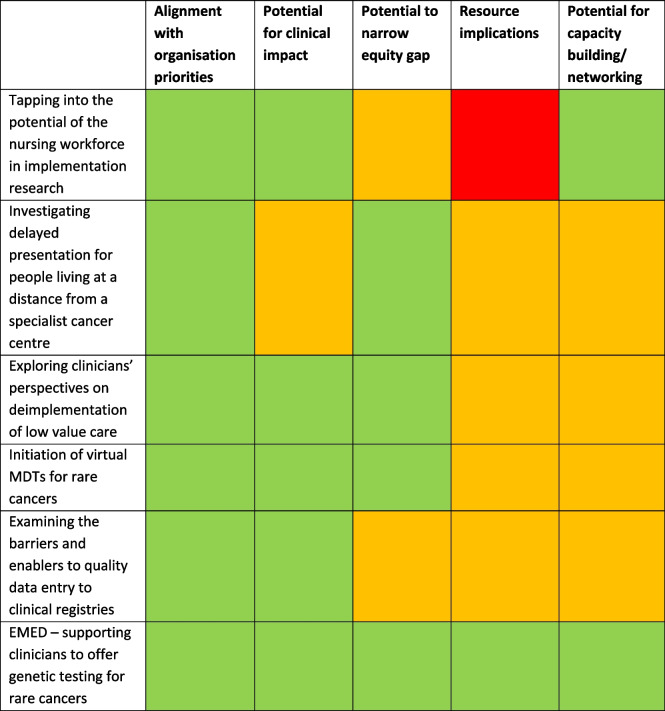
Prioritisation of the projects identified. Key: red – poor; amber – potential; green – good alignment

## Discussion

Our study revealed several potential implementation science priorities for the delivery of cancer care. Four themes were identified that ranged across individual clinical areas to strategic, cross organisation thinking. 1) *Integration of services to address organisational priorities*: This theme reflects a common concern about fragmented services and the impact on the care of patients with cancer [31]. The focus of this first theme is on the strategic level and implementation science can facilitate investigation into which services should be scaled up and/or sustained which ones should be deimplemented to facilitate integration. A critical step here is the development of effective communication structures and promoting team work to support any changes made. A range of tools exist e.g., the Intervention Scalability Assessment Tool (ISAT) [32] and the Program Sustainability Assessment Tool(PSAT) [33] to initiate the planning required and potentially challenging discussions into integration of services. 2) *Application of digital health interventions:* With the speed of growth of digital health interventions it would be easy to lose focus on consumer needs and the importance of equity in delivering cancer care. As a result, uptake can be poor and reflect unwarranted variation which is often associated with inequitable health outcomes [34]. Implementation science can play a role here along the phases of implementation from an initial needs assessment, co-designing an appropriate digital health intervention with all key stakeholders, through to planning for the evaluation of the impact of the intervention [35]. Where these steps are not followed there is a risk of designing digital health interventions that are not desired by the target population, especially subpopulations with greatest need, leading to non-adoption and so contribute to research waste and lack rigorous evaluation of effectiveness, feasibility, acceptability and fidelity central to determining implementability [36]. 3) *Identifying potential for implementation research, including deimplementation*: There was enthusiasm to undertake implementation research though participants highlighted the importance of rigour and quality raising the importance of growing awareness of when implementation science may be of benefit and building implementation research capacity. Frontline clinicians and consumers' experience make them perfectly positioned to recognise care practice gaps though they may not have the implementation science literacy to convert this into implementation research. Recognising a care gap is an essential first step for any implementation science study. Building capacity in implementation science with those who have a frontline role could lead to targeted implementation research in areas of importance to key stakeholders. 4) *Focusing on direct consumer engagement*: The role of consumers in implementation research is pivotal to ensure relevant and meaningful studies are undertaken that respond to consumer needs [14, 15]. While there is a strong focus on consumer engagement at the study organisations (https://vcccalliance.org.au/our-work/consumer-engagement/; https://www.petermac.org/get-involved/consumer-participation) there is a need, similar to clinicians, to develop consumer awareness and familiarity in the role of implementation science leading to increased collaborative studies. A summary of the challenges identified through the study and proposed approaches to overcome these issues can be found in Table 5.

**Table 5:** Summary of issues identified and suggested approaches to address issue

**Issues identified**	**Suggested approaches to address issue**
Fragmented services limiting potential to achieve organisational priorities	Prioritise effective communication structures and team building Identify what services could be scaled up or sustained using a validated tool e.g. Intervention Scalability Assessment Tool and the Program Sustainability Assessment Tool
Effective implementation of digital health interventions	Initial needs assessment Co-design an appropriate digital health intervention with all key stakeholders Plan and deliver evaluation of the impact of the intervention
Lack of implementation science literacy	Build implementation research awareness and capacity with stakeholders including clinicians, consumers and those in high administrative positions such as policy makers.

Six potential pilot implementation research test cases were identified (Table 4) which would have varying impacts and benefits for participants and the potential to engage other researchers such as health economists. The selected pilot Implementation research study, EMBED, provides an opportunity to raise awareness of the role of implementation science in clinical practice and for capacity building. This study has the advantage of both top down organisational leadership support and bottom up clinician and consumer input [8]. It will be critical that we evaluate both the implementation and clinical impacts of undertaking this pilot Implementation research project.

We strengthened the impact of our stakeholder engagement through the use of theory for example in designing the interview schedule [23] and in establishing the prioritisation process [26, 27]. One of the benefits of adopting a theory informed approach is that it builds on what is already known so continues to grow the knowledge base and prevents the risk of reinventing the wheel [37]. The use of theory also enhances the potential for replicability and clarity in research focus with the use of common terminology [38]. Additionally, theory can provide information about why an activity fails or succeeds [39, 40]. In complex organisational settings, such as cancer care, single frameworks at times are insufficient to meet the research question needs. Examples where theories have previously been combined include, the Theoretical Domains Framework, a psychosocial behaviour change framework synthesised from 33 behaviour change theories [41] and the Consolidated Framework for Implementation Research, used to assess barriers and facilitators to implementation, comprised of 19 frameworks [42]. In this scenario of complex health interventions, it can be advantageous to synthesise frameworks to address the challenge presented and a robust and transparent process is required to ensure the synthesis is replicable and relevant.

This study has limitations. This study took place while COVID-19 restrictions were still in place. At times these restrictions curtailed access to study participants leading to a longer data collection period than planned. Study participants were drawn from the PMCC and VCCC Alliance so the priorities identified will be limited these two organisations. We were fortunate to have experienced consumer input from two consumer advocates. Increased and more diverse consumer representation would provide a more comprehensive consumer voice and so further strengthen the relevance of our findings. Finally, a lack of resources limited the selection of a larger number of pilot implementation research studies.

## Conclusion

We have proposed a structured method for aligning implementation research priorities with the strategic priorities of healthcare organisations, to maximise the likelihood that implementation efforts will be focused on activities with the highest potential impact. This study used a combination of stakeholder engagement and organisational/implementation science theory to prioritise an implementation research project that would have the greatest chances of success (i.e. least likely to lead to research waste) by ensuring alignment with stakeholder priorities. Next steps include the design, delivery and evaluation of the EMBED pilot Implementation research project.

### Supplementary Information

**Supplementary Material 1.** 

**Supplementary Material 2.** 

## Data Availability

The datasets generated and/or analysed during the current study are not publicly available due to ethical constraints but are available from the corresponding author on reasonable request.
