# Supplementary material for: Aligning organisational priorities and implementation science for cancer research
Source: BMC Health Serv Res. 2024 Mar 14;24:338. doi: 10.1186/s12913-024-10801-x (PMC10938739; doi:10.1186/s12913-024-10801-x)
Supplement: Supplementary file 1 — Supplementary Material 1. [file 12913_2024_10801_MOESM1_ESM.docx]

**Supplementary information I: Interview guide**

Could you tell me a little about your role at PMCC/VCCC?

Exemplar questions follow…

1. What is the implementation problem you would like to see addressed at PMCC/VCCC?

*Prompt*: Is it focused on,

- Interventions under development
- Slow uptake of a new intervention that is clinically effective
- Unwarranted variation in care that is associated with health inequities
- Premature or continued uptake of an intervention or technology that is now known to be ineffective, wasteful or harmful
- Failure to keep up with changes in evidence relating to an existing intervention
- Need to evaluate an existing implementation intervention(1)

1. Can you describe the healthcare intervention you feel would meet this problem?
2. What evidence do you have that the intervention leads to patient benefit?
3. How do you know this is a problem – what evidence do you have about the care gap?
4. Does this have the potential to be relevant to other sites/clinical specialities? If so, how?
5. Who are the key members of the research team and what are their disciplines?
6. How do you propose implementation science activity is funded?

- Application to a competitive funding round
- Application for funding from a healthcare provider
- Existing funds

1. Any further comments? (e.g., project stage, team availability for meetings, likely impact, previous involvement with implementation science)

1.Patey AM FG, Francis JJ, McCleary N, Presseau J, Grimshaw JM. . Healthcare professional behaviour: Health impact, prevalence of evidence-based behaviours, correlates and interventions. . Psychology & Health In press.
