# Supplementary material for: Aligning organisational priorities and implementation science for cancer research
Source: BMC Health Serv Res. 2024 Mar 14;24:338. doi: 10.1186/s12913-024-10801-x (PMC10938739; doi:10.1186/s12913-024-10801-x)
Supplement: Supplementary file 2 — Supplementary Material 2. [file 12913_2024_10801_MOESM2_ESM.docx]

**Supplementary information II: Interview summary template**

| **Participant ID** |  | **Date** |  | **Affiliations** |  |
| --- | --- | --- | --- | --- | --- |

| **About the implementation challenge/problem** | |
| --- | --- |
| What is the implementation problem you want to address?   - Intervention under development - Slow uptake of a new intervention that is clinically effective - Unwarranted variation in care that is associated with health inequities - Premature or continued uptake of an intervention or technology that is now known to be ineffective, wasteful or harmful - Failure to keep up with changes in evidence relating to an existing intervention - Need to evaluate an existing implementation intervention - Other |  |
| How do you know this is a problem – evidence? |  |
| **About the solution?** | |
| Describe the idea/project |  |
| What evidence is there to support this? |  |
| Have consumers been involved – how? |  |
| Potential for generalisation? |  |
| **Resources** | |
| Team members |  |
| Plans for funding |  |
| **Other comments** | |
|  |  |
